# Surprising longhorned beetle (Coleoptera, Cerambycidae) richness along an Italian alpine valley

**DOI:** 10.3897/zookeys.208.3193

**Published:** 2012-07-17

**Authors:** Mauro Gobbi, Cristiana Priore, Clara Tattoni, Valeria Lencioni

**Affiliations:** 1 Department of Invertebrate Zoology and Hydrobiology, Museo delle Scienze, Via Calepina 14, I-38122 Trento, Italy; 2 Via A. da Taranto 8, 73100, Lecce, Italy; 3 Department of Vertebrate Zoology, Museo delle Scienze, Via Calepina 14, I-38122 Trento, Italy

**Keywords:** Cerambycids, saproxylic, species richness, protected areas, Val Genova, Alps

## Abstract

In this paper we report about 88 longhorned beetles (Cerambycidae) species found in 6929 hectares and distributed along an altitudinal gradient of 1500 m of an Italian alpine valley (Val Genova, central-eastern Italian Alps). The species richness, result merging data from sixty years (1947–2007) of entomological surveys, corresponds to the 32% of the Italian cerambycid fauna confirming the high richness/surface ratio, probably unique in the Alps. The effect of thirteen environmental variables was tested on the species richness, but only the elevation resulted able to affect it. The species richness decrease with altitude not gradually, but experience a strong step above 1700 m a.s.l.. The highest species richness (average values of 42 species) was recorded at the lowest and mid elevations (between 800 and 1600 m a.s.l.). The species turnover along the altitudinal gradient is low suggesting moderate habitat turnover along the valley.

One of the eighty-eight observed species, *Tragosoma depsarium*,is classified near threatened by the IUCN. Our data suggest that the wilderness of the valley close to the suitable management of grasslands and forests, help to support high level of cerambycids diversity. This biodiversity is good indicators of health of the wood saproxylic assemblages, as well an important food source for many vertebrate predators.

## Introduction

The longhorned beetles (Coleoptera: Cerambycidae) can be considered one of the richest families of animals with about 35,000 described species (Hurka 2006). The European species richness amounts to a total of 677 species (Sama 2009). This family of beetles is widespread in all Europe from the Mediterranean basin to the highest latitude due to the ability to colonize from temperate to hot regions. Cerambycids are strictly phytophagous occurring herbaceous, shrubby and arboreal vegetation (Sama 2006).

From the ecological point of view, longhorned beetles might potentially be excellent indicator species of the health of the wood saproxylic assemblages (Dajoz 2000) because of their habitat specificities, and because they are relatively easy to identify (Speight 1989, Sama 2006). The larvae of most longhorned beetles develop within either living or dead wood and feed by mining galleries in the wood. There is a great range in the breadth of host tree species that may used by the larvae of different species (Hanks 1999). This range of habitat specificity within the family extends from monophagous species specialized on a single host tree species to generalist species that can make use of a large variety of trees genera. Many species, at the adult stages, have an important role as pollinators and mainly the larvae, but also the adult, are an abundant component in the diet of forest birds like the woodpeckers. Thanks to these functional roles in the ecosystems, the cerambycids can be considered good indicators on the state of conservation of biodiversity, but also sensible to human ecosystem management (Sama 2006). In Italy the taxonomical and chorological knowledge is satisfactory. At today, for the Italian peninsula are known 274 species belonging to 119 genera representing one of the nations with the highest species richness in Europe (Sama 2009). This richness can be justified by the longitudinal extension of the mainland, the central position within the Mediterranean area and the presence of highly diversified habitats, both from geomorphologic and climatic point of view (Sama 2006).

In the central-eastern Italian Alps there is a valley belonging to the Adamello-Brenta Natural Park (Trentino – Alto Adige Province), named Val Genova which is characterized by the total absence of urbanized areas, and that is characterized by a mosaic of different natural and human-managed habitats which have not suffered modifications for at least one century. The human activities are limited to the hay meadows, pastures and to the cut of some forested areas. This valley had attracted the attention of many entomologists since the middle of the last century. In the last sixty years many surveys was done by different entomologists to catch cerambycids, therefore we merged presence/absence historical data with those collected by us. The obtained database was used to test the following hypothesis: i) the species richness decreases gradually along the altitudinal gradient, ii) the presence of a mosaic of natural and human-managed habitats supports high values of species richness.

## Methods

### Study area

The study area is an alpine valley named Val Genova (46°09'N, 10°40'E). It is located in the central-eastern Italian Alps, in the Trentino - Alto Adige Region, and belongs to the Adamello-Brenta Natural Park. The valley is about 20 Km long, and the area considered for the cerambycids catchment it is distributed along an altitudinal gradient of about 1500 metres (800–2200 m a.s.l.), and inside an area of 6929 hectares. The average precipitation is over 1000 mm, and the most rainy periods are during May and October with precipitation over 100 mm. Different climatic factors determine the vegetation gradient along the valley. At the lowest elevation (800-1000 m a.s.l.) there is dominance of broadleaf woods with *Fagus sylvatica*, *Alnus incana*, *Acer pseudoplatanus*, *Corylus avellana*, *Fraxinus excelsior*, *Carpinus betulus*, *Laburnum anagyroides*, *Betula pendula*, *Salix alba*, *Robinia pseudoacacia*. At the mid-elevations (1100-1800 m a.s.l.) the broadleaf forests (mainly *Betula*) are mixed with conifers characterized by the presence of *Abies excelsa*, *Abies alba* and *Pinus sylvestris*. The timberline is around 1900 m a.s.l., while the treeline (with *Larix decidua*) is around 2100 m a.s.l.. Above 2100 m shrubs of *Alnus viridis*, *Rhododendron ferrugineum* and rare *Pinus mugo* appear. Along this altitudinal and vegetation gradient there are many grasslands (e.g. pastures, meadows) human-managed for at least one century. In particular the hay meadows are located at the low-mid elevation (< 1500 m a.s.l.), whereas the pastures are from the mid to the highest elevation (1300–2200 m a.s.l.). Studies performed in the Adamello-Brenta Natural Park evidenced just an increase of 10% of the forest coverage since the Second World War up today due to the increase of neglected areas, in particular at the highest elevations (Bronzini 2005).

### Database creation

The database has been created by merging data collected during the field surveys performed by Moscardini (1956), Contarini (1988), Sama (1988), Martinelli (1995), and Pedroni (1998, 2000), with our presence/absence data recorded in 2007.

Our survey of longhorned beetles was carried out from May to September 2007, two-three times a week along the bottom of the valley and at the same elevation chosen by Moscardini in 1947 (Moscardini 1956). Other areas located on the slope of the valley were analyzed. To increase the probability of success, the survey of adults was organized on sunny days within the hottest hours (10:00 a.m. – 18:00 p.m.) netting on the herbaceous, shrubby and arboreal vegetation with sweep net and entomological umbrella. Once a week nocturnal collections were made using the Wood’s UV lamp (Southwood 1978).

The surveys performed during these sixty years are not comparable with the aim to describe the species richness trend along the time because the sites visited by each entomologist have been not always the same, and the sampling effort was different.

Longhorned beetles were identified using Pesarini and Sabbadini (1994) and Rastelli et al. (2001). Some specimens were compared with those preserved in the collections of the Museo delle Scienze of Trento (Italy). The updated species nomenclature is based on Sama (2009).

### Statistical analyses

The Incidence-based Coverage Estimator of species richness (ICE) has been used to estimate the gamma-biodiversity (Hortal et al. 2006) of longhorned beetles living in the valley. The species accumulation curve has been drawn using the Mao Tau index to observe how species richness increases with the number of sampling performed by each entomologist (Colwell et al. 2005). The correlation between thirteen different environmental variables (elevation; aspect; distance from: houses, rivers, lakes, main roads, secondary roads; habitats diversity, percentage of: open areas, forests, glades; slope and land cover type) recorded within a buffer of 200 metres around the sampling point, and the species richness (S) has been computed by Spearman’s correlation analyses. This correlation was preferred respect to the Pearson correlation due to the few sampling points. The Linear Regression Analyses has been performed to test the effect of the environmental variables on the species richness. Each environmental variable was calculated by GIS-approach. The ANOSIM test, based on Jaccard’s index of similarity, has been performed to evaluate the presence of similarities in the assemblages’ composition along the elevational gradient. A dendrogram of similarity has been drown to show the similarities along the elevation gradient.

Analyses have been computed using SPSS 13.0 (SPSS, Inc., Chicago IL), PAST 2.0 (http://folk.uio.no/ohammer/past ) and Estimate S (http://viceroy.eeb.uconn.edu/EstimateS ).

## Results

**Figure 1.**
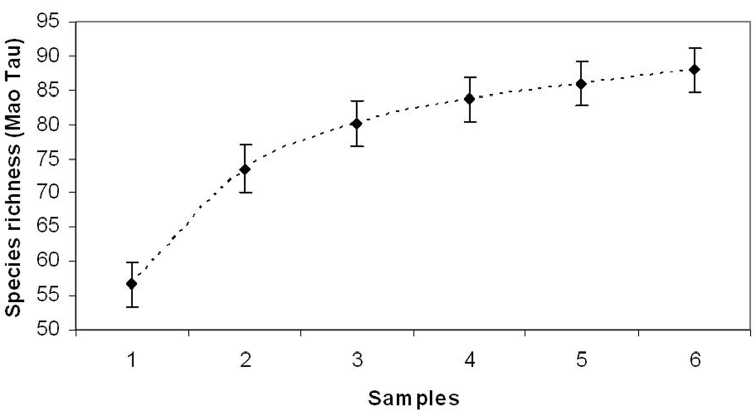
Accumulation curve on the number of species observed during the surveys.

The database realized merging data collected in sixty years (1947-2007) by different entomologists produced a checklist of 88 species (Table 1) observed along the altitudinal gradient comprise between 800 and 2200 m a.s.l.. A new species, never recorded before, has been observed during the last sampling season performed in 2007; the species is *Phytoecia cilindrica* (Linnaeus, 1758). The ICE index estimated for the valley a total of 93 species indicating that about the 91% of the species has been sampled; the species accumulation curve is not tending to the asymptote (Fig. 1), but it is gradually increasing confirming that more species could be cached, yet.

**Table 1.** Cerambycids observed along the altitudinal gradient of the Val Genova (* = presence) The species are ordered on the base of their frequency at each elevational sampling point. (IUCN abbreviations: LC = least concern; NT = near threatened; EU endem = European endemism).

**Species / Elevation (m)**	**800**	**900**	**1060**	**1100**	**1250**	**1430**	**1500**	**1640**	**1790**	**2000**	**2200**	**IUCN Red List**
*Pachytodes cerambyciformis* (Schrank, 1781)	*	*	*	*	*	*	*	*	*	*		
*Tetropium castaneum* (Linné, 1758)	*	*	*	*	*	*	*	*	*	*		
*Monochamus sutor* (Linné, 1758)	*	*	*		*	*	*	*	*	*		LC
*Alosterna tabacicolor* (De Geer, 1775)	*	*	*	*	*	*	*	*				
*Anastrangalia dubia* (Scopoli, 1763)	*	*	*	*	*	*	*	*				
*Anastrangalia sanguinolenta* (Linné, 1758)	*	*	*	*	*	*	*	*				
*Evodinus clathratus* (Fabricius, 1792)			*	*	*	*	*	*	*	*		
*Leptura quadrifasciata* (Linné, 1758)	*	*	*	*	*	*	*	*				
*Monochamus sartor* (Fabricius, 1787)	*	*		*	*	*	*	*	*	*		LC - European endemism
*Rhagium inquisitor* (Linné, 1775)	*	*	*	*	*	*	*	*				
*Ruptela maculata* (Poda, 1761)	*	*	*	*	*	*	*	*				
*Saperda scalaris* (Linné, 1758)			*	*	*	*	*	*	*	*		LC
*Stenostola dubia* (Laichrting, 1784)		*	*	*	*	*	*	*	*	*		
*Stenurella melanura* (Linné, 1758)	*	*	*	*	*	*	*	*				
*Acmaeops pratensis* (Laicharting, 1784)	*		*	*	*	*	*	*				
*Aromia moschata moschata* (Linné, 1758)		*	*	*	*	*	*	*				LC
*Callidium violaceum* (Linnaeus, 1758)				*	*	*	*	*	*	*		LC
*Gaurotes virginea* (Linné, 1758)	*	*	*	*	*	*		*				
*Hylotrupes bajulus* (Linné, 1758)	*	*	*	*	*	*	*					LC
*Lepturobosca virens* (Linné, 1758)			*	*	*	*	*	*	*	*		
*Oxymirus cursor* (Linné, 1758)			*	*	*	*	*	*	*		*	
*Agapanthia villosoviridescens* (De Geer, 1775)			*	*	*	*	*	*				
*Judolia sexmaculata* (Linné, 1758)			*	*	*	*	*	*			*	
*Molorchus minor* (Linné, 1758)			*	*	*	*	*	*				LC
*Paracorymbia hybrida* (Rey, 1885)			*	*	*	*	*	*				
*Paracorymbia maculicornis* (De Geer, 1775)			*	*	*	*	*	*				
*Pidonia lurida* (Fabricius, 1776)			*	*	*	*	*	*				
*Rhagium bifasciatum* (Fabricius, 1775)			*	*	*	*	*	*				
*Stenocorus meridianus* (Linné, 1758)	*	*	*	*	*	*						
*Tetropium fuscum* (Fabricius, 1787)			*		*	*	*	*	*	*		
*Achanthoderes clavipes* (Schrack, 1781)		*	*	*	*	*						
*Anastrangalia reyi* (Heyden, 1889)				*	*	*	*	*				
*Clytus arietis* (Linné, 1758)	*		*	*	*	*						LC
*Pachyta quadrimaculata* (Linné, 1758)	*	*		*	*	*						
*Stenurella bifasciata* (Muller, 1776)	*	*	*	*	*							
*Tetropium gabrieli* (Weise, 1905)				*	*	*	*	*				
*Cortodera femorata* (Fabricius, 1787)		*			*	*		*				
*Pseudalosterna livida* (Fabricius, 1776)			*	*	*	*						
*Rhagium mordax* (De Geer, 1775)			*	*	*	*						
*Stictoleptura rubra* (Linné, 1758)	*	*		*	*							
*Anaglyptus mysticus* (Muller, 1766)		*			*	*						LC
*Asemum striatum* (Linné, 1758)					*	*		*				
*Dinoptera collaris* (Linné, 1758)	*	*	*									
*Lamia textor* (Linné, 1758)	*	*	*									
*Obrium brunneum* (Fabricius, 1792)	*		*		*							LC
*Pachyta lamed* (Linnè, 1758)						*	*	*				
*Pogonocherus hispidulus* (Piller & Mitterpacher, 1783)	*	*	*									
*Prionus coriarius* (Linné, 1758)	*	*	*									LC
*Saphanus piceus* (Licharting, 1784)			*	*	*							
*Spondylis buprestoides* (Linné, 1758)	*	*	*									
*Strangalia attenuata* (Linné, 1758)	*	*	*									
*Tragosoma depsarium* (Linné, 1767)						*	*	*				NT
*Cerambyx scopolii* Fuesslins, 1775	*					*						LC
*Cholorophorus figuratus* (Scopoli, 1763)	*					*						LC
*Clytus lama* (Mulsant, 1847)	*					*						LC - European endemism
*Exocentrus punctipennis* Mulsant & Guillebeau, 1856	*	*										
*Leiopus nebulosus* (Linné, 1758)					*	*						
*Mesosa nebulosa* (Fabricius, 1781)	*	*										
*Oberea pupillata* (Gyllenhal, 1817)						*		*				
*Paracorymbia fulva* (De Geer, 1775)	*	*										
*Parmena unifasciata* (Rossi, 1790)	*	*										
*Phytoecia cylindrica* (Linnaeus, 1758)	*	*										
*Phytoecia nigricornis* (Fabricius, 1781)	*	*										
*Pogonocherus fasciculatus* (De Geer, 1775)			*					*				
*Saperda carcharias* (Linné, 1758)	*	*										
*Stenopterus rufus* (Linné, 1767)	*	*										LC
*Stenostola ferrea* (Schrank, 1776)	*					*						
*Acmaeops septentrionis* (Thompson, 1666)					*							
*Aegomorphus clavipes* (Schrank, 1781)	*											
*Anoplodera rufipes* (Schaller, 1783)			*									
*Anoplodera sexguttata* (Fabricius, 1775)		*										
*Arhopalus ferus* (Mulsant, 1839)		*										
*Arhopalus rusticus* (Linné, 1758)			*									
*Brachyta interrogationis* (Linné, 1758)									*	*		
*Callidium aeneum* (De Geer, 1775)					*							LC
*Cholorophorus sartor* (Muller, 1766)		*										LC
*Corymbia scutellata scutellata* (Fabricius, 1781)			*									
*Exocentrus lusitanus* (Linné, 1767)	*											
*Glaphyra umbellatarum* (Schreber, 1759)						*						LC
*Grammoptera ruficornis* (Fabricius, 1781)		*										
*Mesosa curculionoides* (Linné, 1758)	*											
*Oberea oculata* (Linné, 1758)	*											
*Oplosia cinerea* Mulsant, 1839 (=fennica Paykull, 1800)					*							
*Saperda octopunctata* (Scopoli, 1772)			*									LC
*Saperda populnea* (Linnè; 1758)								*				
*Stenopterus ater* (Linné, 1767)		*										LC
*Stenurella nigra* (Linnè, 1758)	*											
**alpha diversity**	43	41	46	38	48	49	32	37	12	11	2	

**Figure 2.**
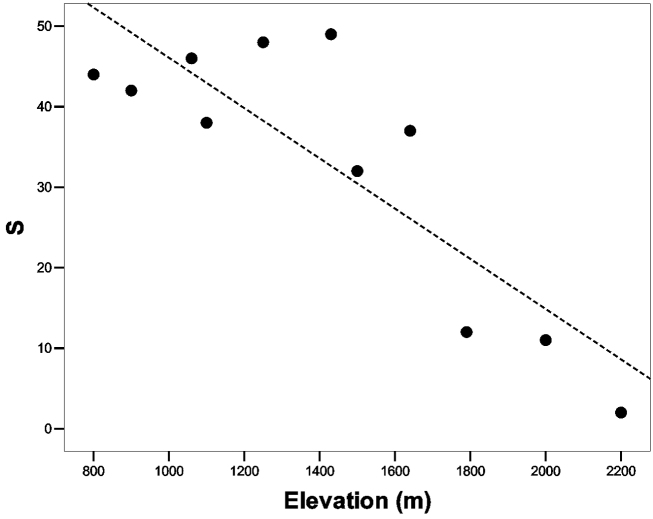
Relationship between the species richness (S) and the altitudinal gradient.

The Spearman’s correlation analysis highlighted that species richness is correlated to two variables: the elevation (rho = -0,74; P = 0,01), and of the distance from the secondary roads (rho = -0,65; P = 0,03), but elevation and the distance from the secondary roads are positively auto-correlated (rho = 0,73; P = 0,01). The Linear Regression Analyses performed to test the effect of elevation and distance from the secondary roads on the species richness show that elevation is the only variable able to affect the negatively the specie richness (ANOVA test: F_2,10_ = 10,27; P = 0,006; elevation: t = -2,58; P = 0,033; distance from the secondary roads: P = 0,95). In particular, species richness decreased with increasing elevation, but this trend is not gradual; the highest values of S are between 800 and 1600 m a.s.l. (S_mean_ = 41,14). Within this elevational gradient, any significant trend in the species richness resulted (P = 0,45) (Fig. 2).The species richness decrease strongly above 1600 metres, and between 1700 and 2200 metres; the average species richness is 8.

**Figure 3.**
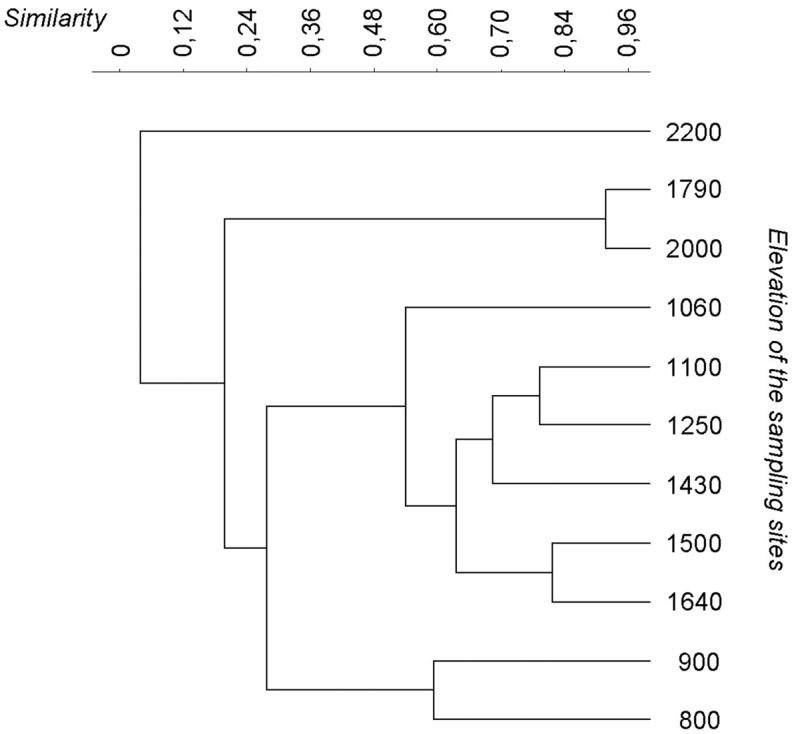
Dendrogram drawn on the base of the Jaccard similarities between the sites.

ANOSIM test demonstrated that species composition along the valley varied with a significantly low turnover (ANOSIM, r = 0.21; P < 0.01). The dendrogram built on the base of the Jaccard similarity showed the cluster of three main groups (Fig. 3): the first one is between 800 and 900 m asl, the second one is between 1060 and 1640 m, and the third is above 1790 m a.s.l..

## Discussion

The high species richness (S = 88) found in Val Genova valley reflect the high wilderness of this area due to the cerambycids being excellent indicators of the health of the wood decomposer community because of their habitat specificities (Nilsson et al. 2001). This surprising species richness, found in 69,3 km², corresponds to the 60% of the Trentino – Alto Adige Region (13607 km²) cerambicid fauna, to the 32% of the Italian and the 13% of that European one (Fauna Europaea 2011). Probably the agrosilvopastural management conduced during the decades has been not extensive like as happened in similar areas of the Alps. The agrosilvopastural management adopted in the studied valley is able to prevent, or at least to minimize, the forest colonization in the neglected grasslands and aimed to maintain into the forest patches with necromass stocks able to attract the cerambycid decomposers community.

The effects of the environmental variables on the species richness showed that only the elevational gradient determines a decrease in the number of species, and a low spatial assemblage’s turnover. This result is in agreement with Baselga (2008) who highlighted that species richness gradient is a phenomenon more environmentally deterministic than turnover that is independent of the richness gradient. The species richness trend was not gradual, as expected, but affected by a step around 1700–1800 metres. This step coincides with the timberline. So, under the timberline, the species richness maintains high values due to the presence of a spatially structure vegetation, and by the mosaic of agrosilvopastural activities.

Twenty-five percent of the species observed in the Val Genova have been evaluated in the IUCN redlist of saproxylic beetles (Nieto and Alexander 2010) and have been considered species of “least concern” (LC), but within these species *Tragosoma depsarium* is considered “near threatened” (NT), and two (*Monochamus sartor* and *Clytus lama*) are endemic of Europe (Table 1). Besides, within these species of European conservation interest, there are some of local monitoring interests because they have not been observed anymore since at least twenty years, therefore reported for the last time by Contarini (1988), and Sama (1988). These species are eight, and specifically: *Parmena unifasciata*, *Grammoptera ruficornis*, *Oberea oculata*, *Anoplodera sexguttata*, *Arhopalus ferus*, *Arhopalus rusticus*, *Paracorymbia fulva*, and *Phytoecia nigricornis*.

The forest management is known to negatively affect saproxylic beetles (Paillet et al. 2010). This finding suggests that the forests on the Val Genova valley are not extensively exploited by humans (mainly the thinning and the clearing) favouring the amount of dead and stressed trees. Raje et al. (2011) reports that the forest productivity is negatively correlated with cerambycid richness. A decrease in the productivity favour availability of dead wood, fallen limbs and more exposed inner wood offering favourable conditions for the development of the cerambycids. These beetles represent an important food source for many vertebrate and invertebrate predators, such that the benefits could propagate through the food web.

In conclusion, the biodiversity of longhorned beetles observed in the Val Genova valley can be considered surprising due to the high number of species living into an area with the following features: small size and distributed along a wide elevational gradient.

The possibility to observe so many cerambycids in a limited space is to our knowledge, unique in the Alps. It suggest that other taxonomic groups should be considered with the purpose to increase the entomological knowledge of this valley that probably is the only one not urbanized, at least in the Italian Alps.
